# High-Throughput Sequencing to Reveal Genes Involved in Reproduction and Development in *Bactrocera dorsalis* (Diptera: Tephritidae)

**DOI:** 10.1371/journal.pone.0036463

**Published:** 2012-05-03

**Authors:** Weiwei Zheng, Tao Peng, Wei He, Hongyu Zhang

**Affiliations:** 1 State Key Laboratory of Agricultural Microbiology, Hubei Key Laboratory of Insect Resource Application and Sustainable Pest Control and Institute of Urban and Horticultural Pests, College of Plant Science and Technology, Huazhong Agricultural University, Wuhan, Hubei, People's Republic of China; 2 Shanghai Hanyu Bio-Lab, Shanghai, People's Republic of China; Karlsruhe Institute of Technology, Germany

## Abstract

**Background:**

Tephritid fruit flies in the genus *Bactrocera* are of major economic significance in agriculture causing considerable loss to the fruit and vegetable industry. Currently, there is no ideal control program. Molecular means is an effective method for pest control at present, but genomic or transcriptomic data for members of this genus remains limited. To facilitate molecular research into reproduction and development mechanisms, and finally effective control on these pests, an extensive transcriptome for the oriental fruit fly *Bactrocera dorsalis* was produced using the Roche 454-FLX platform.

**Results:**

We obtained over 350 million bases of cDNA derived from the whole body of *B. dorsalis* at different developmental stages. In a single run, 747,206 sequencing reads with a mean read length of 382 bp were obtained. These reads were assembled into 28,782 contigs and 169,966 singletons. The mean contig size was 750 bp and many nearly full-length transcripts were assembled. Additionally, we identified a great number of genes that are involved in reproduction and development as well as genes that represent nearly all major conserved metazoan signal transduction pathways, such as insulin signal transduction. Furthermore, transcriptome changes during development were analyzed. A total of 2,977 differentially expressed genes (DEGs) were detected between larvae and pupae libraries, while there were 1,621 DEGs between adults and larvae, and 2,002 between adults and pupae. These DEGs were functionally annotated with KEGG pathway annotation and 9 genes were validated by qRT-PCR.

**Conclusion:**

Our data represent the extensive sequence resources available for *B. dorsalis* and provide for the first time access to the genetic architecture of reproduction and development as well as major signal transduction pathways in the Tephritid fruit fly pests, allowing us to elucidate the molecular mechanisms underlying courtship, ovipositing, development and detailed analyses of the signal transduction pathways.

## Introduction

Tephritid fruit flies are of major economic importance in agriculture, causing damage to fruits and other plant crops. The genus *Bactrocera* is of worldwide notoriety for its destructive impact on agriculture. Adults lay eggs in fruit and the hatched larvae feed inside. Various species of fruit flies in the genus *Bactrocera* vary in the number of generations and in the type of host plants used for feeding and ovipositing. Due to the wide host range and high fecundity of the adults as well as the great adaptability of the larvae, effective control of *Bactrocera* is of great value. The oriental fruit fly, *B. dorsalis*, is one of the most important quarantine pests in Asian countries. It can feed on up to 250 different types of fruits and vegetables, causing severe ecnomic loss [1]. Therefore, it is important to identify more molecular targets involved in adult reproduction and larval development in *B. dorsalis*, to formulate simple and effective strategies in agricultural pest control.

Compared to the model insect species whose genomes have been sequenced, such as *Drosophila melanogaster*, *Anopheles gambiae*, and *Bombyx mori*, genomic sequence resources for Tephritid fruit flies are limited. The EST approach to obtain detailed information on transcriptome signatures that relate to some physiological processes in the medfly *Ceratitis capitata* has indicated the significance of large-scale gene discovery in insects lacking genomic sequence information [2]. But gene discovery using 3730 DNA analyzer in the *C. capitata* cDNA libraries of embryos and adult heads is limited to genes involved in reproduction, sex determination, and chemosensory perception [2]. Although the known resources available on NCBI for *B. dorsalis* have facilitated the study of gene function during yolk protein synthesis [3], [4], large scale molecular studies in *B. dorsalis* remain limited. There is a lack of large-scale sequencing in species of Tephritidae.

The introduction of novel high-throughput sequencing technologies has provided significant convenience for further studies of non-model organisms including insects [5], [6]. Next generation sequencing technologies such as the 454 pyrosequencing and Illumina have been widely used to identify genes involved in several physiological processes and behaviors. An EST library has been built for the flesh fly *Sarcophaga crassipalpis* utilizing massively parallel pyrosequencing with the Roche 454-FLX platform [7]. A transcriptomic study in the lepidopteran model host *Galleria mellonella* by 454 pyrosequencing has created extensive resources for *Galleria*, especially for immune related genes [8]. The 454 sequencing technology has also been used in the milkweed bug *Oncopeltus fasciatus* to discover genes participating in several early developmental processes [9]. The transcriptome of *Bemisia tabaci* has been built by Illumina sequencing and a large number of genes associated with development and insecticide resistance were identified [10]. Transcriptome analysis of *B. dorsalis* which mainly focused on genes involved in insecticide resistance has been performed by Illumina sequencing recently [11]. However, a comprehensive identification of genes involved in reproduction and development as well as major signal transduction pathways in *B. dorsalis* remains unavailable.

In the present study, we present the results from the sequencing and assembly of the transcriptome of *B. dorsalis* (whole body) at different developmental stages (larvae from three instars, pupa and adult) using the 454-pyrosequencing technology. Genes involved in reproduction and development as well as nearly all major conserved metazoan signal transduction pathways were largely identified. Additionally, a great number of differentially expressed genes were obtained and functionally annotated, and the gene expression patterns for some of these genes were verified by qRT-PCR. This transcriptome is undoubtedly valuable for molecular studies of the underlying mechanisms in reproduction and development and is a useful resource for further exploring signal transduction pathways. Furthermore, our data will provide insights for the development of effective and eco-friendly pest control strategies.

## Results and Discussion

### Generation and assembly of oriental fruit fly ESTs

To obtain an overview of the *B. dorsalis* gene expression profile during development, cDNA samples from different developmental stages (Table 1) were prepared and sequenced on a 454 GS-FLX machine. A total of 350,865,036 bases from 747,206 sequence reads with a mean read length 382 bp were obtained (Table 2, Figure S1). The number of reads from the larval, pupal and adult samples was 343,907 reads, 355,839 reads, and 217,426 reads, respectively, with a mean read length of 398 bp, 380 bp, and 359 bp, respectively (Figure S1).

**Table 1:** RNA extraction strategy.

Larval stages	Pupal stages	Adult stages
First instar larvae	Prepupa	Newly emerged adult
Second instar larvae (feeding, molting)	New pupa (2 d after pupation)	Mature adults before mating (sex ratio 1∶ 1)
Third instar larvae (metamorphosing, wandering)	Mid pupa	Mature adults after mating (sex ratio 1∶ 1)
	Old pupa (2 d before eclosion)	

RNA from each life stages were individually extracted and combined equally prior to cDNA library synthesis.

**Table 2:** Summary of the *B. dorsalis* transcriptome.

Total base pairs (bp)	350,865,036
High-quality reads	747,206
Number of reads assembled in contigs (Larvae/Pupa/Adult)	275,375/320,113/151,718
Average read length (bp)	382
Number of contigs	28,782
Average contig length (bp)	750
Range of contig length (bp)	43–11,208
Number of singletons	169,966
Unigenes with E≤0.0001 vs. NR/NT database	48,876/3,744
Estimated unique transcript	134,169

**Figure 1:**
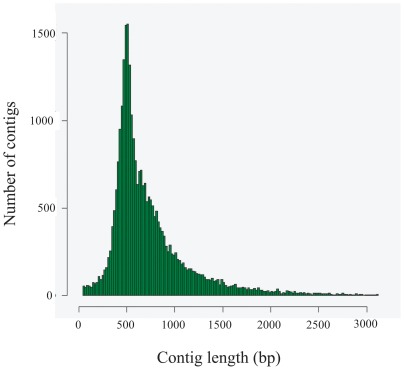
Assembled contig length distribution of the *B.dorsalis* transcriptome. The x-axis indicates contig size and the y-axis indicates the number of contigs of each size.

These raw data were assembled into 28,782 contigs and 169,966 singletons, and finally we generated 48,876 unigenes. The mean contig size was 750 bp with lengths ranging from 43 bp–11,208 bp (Table 2, Figure 1). The contig size distribution revealed the following: more than half of the contigs (14,912; 51.81%) were between 500 and 1000 bp in length; 30.14% (8,676) were less than 500 bp; 17.48% (5,030) of contigs were between 1000 and 3000 bp; and 0.57% (165) were more than 3000 bp (Figure 1). The previously reported *B. dorsalis* transcritome by Illumina sequencing generated 49,804 unigenes with the mean size of 456 bp, and less than 10% (4,404) of unigenes was more than 1000 bp [11]. Therefore, compared with the previous report, our new transcriptome by 454-pyrosequencing makes more detailed and general genetic data available that will facilitate large-scale discovery and utilization of genetic resources for *B. dorsalis*.

To demonstrate the quality of our sequencing data and sequence assembly, 5 contigs (>750 bp) and 5 singletons were randomly selected for RT-PCR analysis. Agarose gel electrophoresis results showed that 9 out of 10 primer pairs resulted in a product of the expected size, and the PCR products were further confirmed by Sanger sequencing (data not shown).

### Annotation of predicted proteins

BLASTX alignments (E-value cut-off of 10^−4^) between the predicted protein sequences and several protein databases, including GenBank non-redundant (NR) and Swiss-Prot, showed that a total of 11,859 (35.8%) predicted proteins could be annotated with known biological functions, whereas the remainder will require more genetic data, which is currently lacking in the fruit fly.

**Figure 2:**
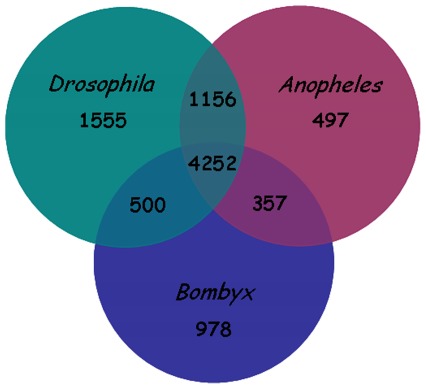
Orthologous genes shared between *Bactrocera*, *Drosophila*, *Anopheles*, and *Bombyx*. The Venn diagram shows the number of orthologous groups of genes shared between the genomes/transcriptomes of these species.

To analyze which part of the assembled sequences had counterparts in certain insect species, orthologous genes shared between *B. dorsalis* and other three insect model species were compared, including either Dipteran *D. melanogaster*, Dipteran *A. gambiae*, or Lepidopteran *B. mori*. We used our *B. dorsalis* ESTs as a query in a BLASTn search (E-value cut-off of 10^−10^) against the databases flybase, vectorbase and silkbase. Given that p values are influenced by the size of the database, a score value of 150 were chosen for the exclusion criteria to make BLAST hits between databases comparable. The results showed 9,295 hits, more than 4,000 of which are common to all insect species and therefore belong to the core invertebrate genes (Figure 2). There were 7,463 identifiable genes shared between *Drosophila* and *Bactrocera* indicating a good coverage of the *Bactrocera* transcriptome, as that many other genes are species-specific. Homologous genes shared between *Anopheles* and *Bactrocera* were 6262, and the number was 6087 between *Bombyx* and *Bactrocera*. Both *B. dorsalis* and *D. melanogaster* belong to Aristocera within Diptera, while *A. gambiae* belongs to Nematocera within Diptera, so *B. dorsalis* and *D. melanogaster* in relationship are near with each other while *A. gambiae* is far with them. It is unsurprising that the number of homologous genes shared between *B. mori* and *B. dorsalis* is the least because Lepidopteran *Bombyx* is in the farthest relation to Dipteran *Bactrocera*.

**Figure 3:**
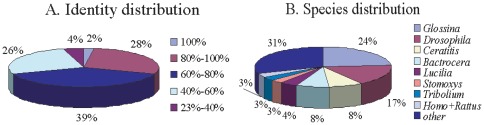
Characteristic analysis of the homology search of ESTs against the nr database. (A) Identity distribution of the top BLAST hits for each sequence. (B) Species distribution is shown as a percentage of the total homologous sequences with an E-value of at least 1.0E^−5^. The first hit of each sequence was used for analysis. Homo: *Homo sapiens*; Rat: *Rattus norvegicus*.

The identity distribution and species distribution were then analyzed (Figure 3). For the identity distribution of the predicted proteins, most of the hits (39%) had 60% to 80% identity with other insects in the nr database, whereas 30% of the sequences had greater identity than 80% (Figure 3A). The species distribution of the top BLAST hits against the nr database for the *Bactrocera* transcriptome showed that *Bactrocera* genes had the greatest number of matches with *Glossina* and *Drosophila* genes. Among them, 24% of the distinct sequences had first hits with sequences from the Dipteran species *Glossina morsitans morsitans* and 17% with sequences from another Dipteran species *Drosophila*, followed by other species within Diptera, including *Ceratitis* (8%), *Bactrocera* (8%), *Lucilia cuprina* (4%) and *Stomoxys calcitrans* (3%) (Figure 3B). The other sequences had hits with other species, such as Coleopteran *Tribolium castaneum* (3%), higher mammalian species *Rattus norvegicus* and *Homo sapiens* (3%).

### Gene ontology and clusters of orthologous groups classification

**Figure 4:**
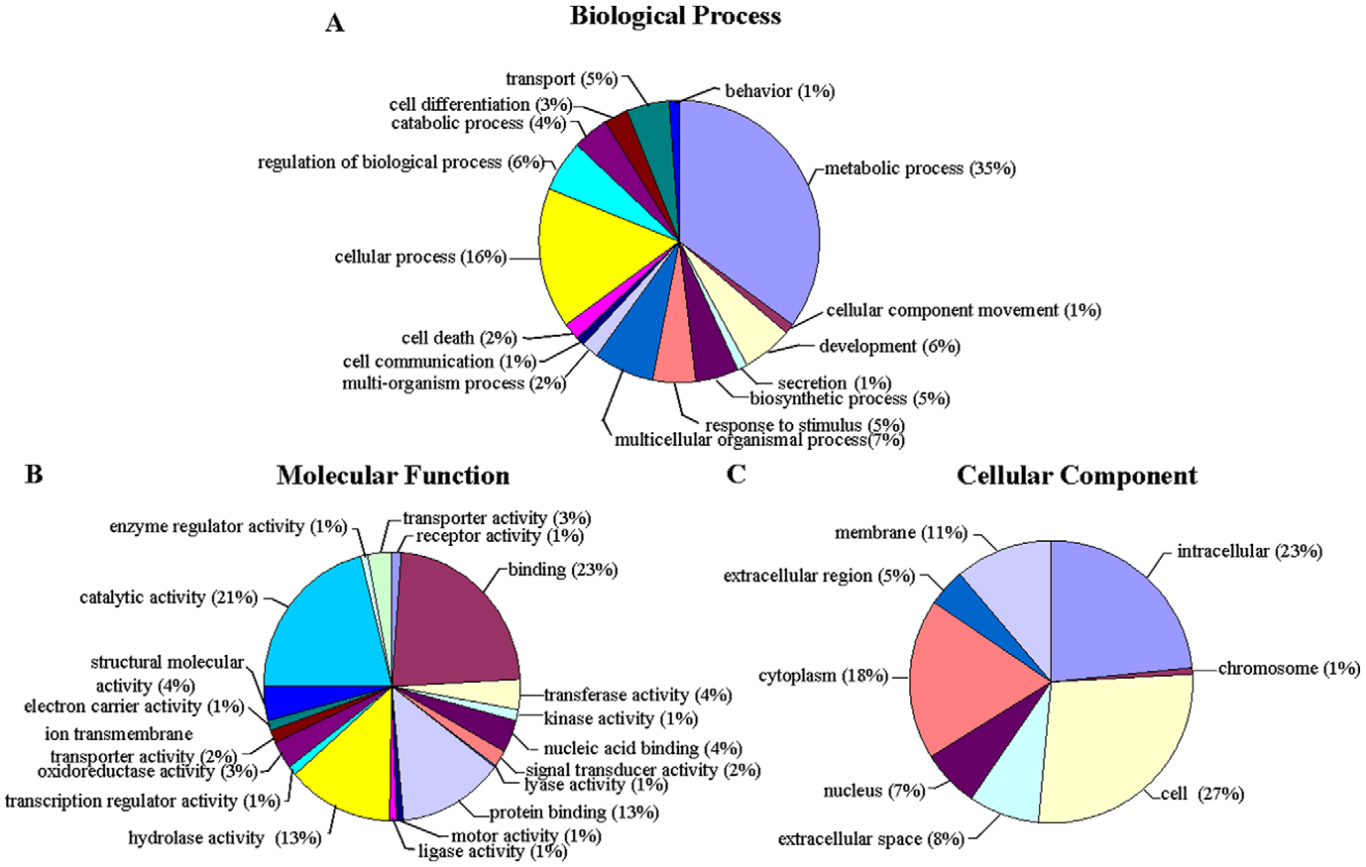
GO analyses of *Bactrocera* transcriptome data. GO analysis of *Bactrocera* sequences corresponding to a total of 28,782 contigs that are predicted to be involved in biological processes (A) and molecular functions (B) and cellular component (C). Classified gene objects are depicted as percentages (in brackets) of the total number of gene objects with GO assignments.

Gene ontology (GO) assignment programs were utilized for functional categorization of annotated genes. In many cases, multiple terms were assigned to the same transcript. These sequences were categorized into 43 main functional groups belonging to 3 categories, including biological process, molecular function, and cellular component (Figure 4). Among the biological processes, the dominant GO terms were grouped into either metabolic (35%) or cellular (16%) (Figure 4A). Within the molecular function category, there was a high-percentage of genes with binding (23%), catalytic activity (21%), protein binding (13%), and hydrolase activity (13%) (Figure 4B). For cellular components, those assignments were mostly given to cell (27%), intracellular (23%), and cytoplasm (18%) (Figure 4C). This GO assignment result is similar to the previously sequenced *B. dorsalis* transcriptome, in which binding, cell and metabolic process were the three largest groups [11].

**Figure 5:**
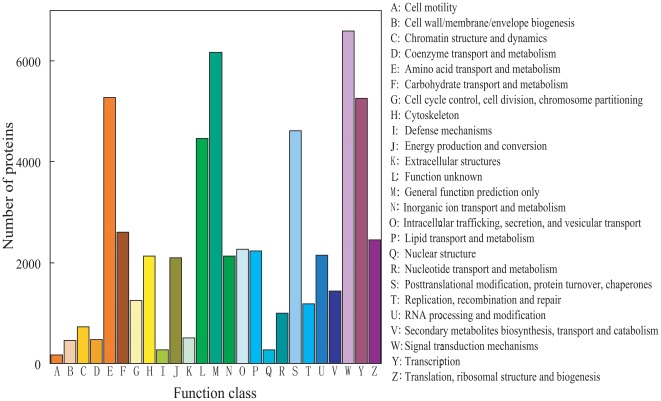
Histogram of clusters of orthologous groups (COG) classification. A total of 58,199 predicted proteins have a COG classification among the 25 categories.

To further evaluate the completeness of our transcriptome library and the effectiveness of our annotation process, assignments of clusters of orthologous groups (COG) were used. Overall, 58,199 proteins were classified as involved in different processes (Figure 5). Among the 25 COG categories, the majority of the clusters were “Signal transduction mechanisms" (6,595, 11.33%), “General function prediction only" (6,171, 10.60%), “Amino acid transport and metabolism" (5,283, 9.08%) and “Transcription" (5,263, 9.04%), whereas “Cell motility" (165, 0.28%), “Defense mechanisms" (272, 0.47%) and “Nuclear structure" (272, 0.47%) represented the smallest groups (Figure 5). Our new sequencing generated a much larger group of genes involved in “Signal transduction mechanisms" than that in the previous report by Shen et al. (2011). In their sequenced *B. dorsalis* transcriptome by Illumina, “General function prediction only" (2,327, 16.49%) was the largest group, followed by “Translation, ribosomal structure, and biogenesis" (1,158, 8.21%), “Transcription" (1,074, 7.61%), “Carbohydrate transport and metabolism" (1,039, 7.36%), whereas “Nuclear structure" was the smallest group (8, 0.057%) [11].

### Genes involved in reproduction

order species.

From the *B. dorsalis* transcriptome, 14 genes have been identified with significant hits to 14 different Dipteran genes known to be involved in reproduction by BLASTX analyses (Table 3). These genes can be divided into 3 classification groups. For the first group, a total of 7 genes are predicted to be involved in male courtship behavior. In *Drosophila*, mutations in the *takeout gene* lead to little or no courtship behavior, and mutations in the *technical knockout gene* cause unsuccessful male courtship behavior [12], [13]. The *quick-to-court gene*, which encodes a predicted coiled-coil protein mainly expressed in the olfactory organs, central nervous system, and male reproductive tract, plays important roles in male courtship behavior. Mutations in this gene cause abnomal male-male or accelerated male-virgin female courtship [14]. We also found a gene homologous to the *Drosophila timeless* in this transcriptome. Copulation times of males are extended in *timeless* mutants [15]. Males carrying mutations in *lingerer*, which is involved in initiation and termination of copulation, disengage their genitalia abnormally [16]. Mutations in *calcium calmodulin kinase II* disrupt the ability of the male to learn to avoid courting males and mated females [17]. The *ken and barbie gene* encodes a putative transcription factor, and mutants feature malformation of terminalia in adult *Drosophila* [18].

**Table 3:** Oriental fruit fly assembled sequences with best-hit matches to Dipteran genes involved in reproductive behaviors.

Gene ID	Dipteran gene	Length (bp)	E-value	Protein Identity (%)	Function (Species)
**Group I**					male courtship
1-G0EDD0L01DWQ2X	*calmodulin*	551	8e-98	100	*D. melanogaster*
contig2395	*takeout*	449	1e-47	58	*G.. morsitans*
Contig10357	*technical knockout*	649	8e-67	76	*D. melanogaster*
Contig1089	*timeless*	1073	3e-103	83	*D. melanogaster*
Contig20167	*ken and barbie*	778	1e-69	89	*D. yakuba*
Contig363	*lingerer*	2122	3e-31	54	*D. melanogaster*
contig8597	*quick to court*	948	2e-74	92	*D. melanogaster*
**Group II**					female fertility, courtship
1-G0EDD0L01D0M5P	*sex peptide receptor*	406	4e-66	75	*D. melanogaster*
contig27364	*logjam*	1015	9e-129	84	*D. melanogaster*
Contig23838	*sphingosine kinase 2*	457	4e-08	43	*D. melanogaster*
Contig934	*forkhead box O*	1133	1e-80	83	*D. melanogaster*
1-G0EDD0L01EQVP9	*forkhead box L*	431	6e-24	70	*A. aegypti*
1-G0CLBUI04IB3H6	*forkhead box P*	342	9e-08	43	*D. melanogaster*
**Group III**					sexual orientation, courtship
1-G0CLBUI04IPTV3	*fruitless*	434	2e-35	62	*D. heteroneura*

*A. aegypti: Aedes aegypti*; *D. melanogaster: Drosophila melanogaster*;

*G.. morsitans: Glossina morsitans morsitans.*

Sequences in the second group share a high degree of homology with genes involved in female reproduction in *Drosophila* and mosquitoes, including the *Drosophila logjam*, *sphingosine kinase 2*, *forkhead box*, and *sex peptide receptor (SPR)*. The *logjam* gene encodes a predicted protein homologous to the EMP24/GP25 transmembrane components of cytoplasmic vesicles and may participate in the intracellular trafficking of positive signals for oviposition. Mutants for this gene are unable to oviposit mature eggs but display normal courtship behavior and fertility [19]. Mutations in *sphingosine kinase 2* have no effect on viability at any developmental stage or on adult longevity but do result in the reduction of flight ability and fecundity, which might be caused by retention of mature eggs in the ovaries [20]. The forkhead-box (Fox) proteins constitute a large and diverse group of transcription factors characterized by a conserved 110-amino acid ‘Fox’ DNA-binding domain. Fox genes that belong to various subgroups (A-Q) have been found to be involved in many biological processes, including development, metabolism, and immunoregulation [21]–[25]. Three Fox gene homologs were found in the *B. dorsalis* transcriptome, including *FoxL*, *FoxO* and *FoxP*. Fox transcription factors reportedly play important roles in regulating reproduction in *Aedes aegypti*. Silence of mosquito *FoxL* and *FoxO* reduces the amino acid-induced vitellogenin gene expression, which leads to fewer eggs laid [26]. Another female reproductive gene is *SPR*, which mediates the post-mating switch in reproductive behaviour of female *Drosophila*. Knockdown of *SPR* results in very few eggs laid after mating, and RNAi-treated females remate frequently [27].

Notably, *fruitless (fru)* belongs to the third group. It can be processed into male-specific or female-specific proteins by alternative splicing [28], [29], orchestrating both male courtship behavior and sexual orientation [28]–[32].

### Genes involved in signal transduction

Genes involved in signal transduction pathways have been listed in Table S1. The results showed that “Signal transduction mechanisms" constitutes the majority of the clusters within the metabolism pathway classification of the *B. dorsalis* transcriptome (Figure 5), including hormone signaling, insulin signaling, MAPK, Wnt, Notch, and Hedgehog (Table S1).

Among these signaling pathways, the insulin signaling pathway is one of the most important. Insect growth and developmental processes, among which molting and metamorphosis are the most important physiological events, are regulated by two major hormones: steroid 20-hydroxyecdysone (20E) and sesquiterpenoid juvenile hormone (JH). Both the 20E and JH signal transduction pathways have been well studied [33]–[37]. Recently, another conserved signaling pathway called the insulin signaling pathway has been studied in *Drosophila* and has been demonstrated to play an essential role in controlling insect body, organ, and cell size. Several protein are involved in this pathway, including PI(3)K, PTEN, and Akt/PKB [38]–[40].

16 genes have been identified to share high homology with insect genes known to be involved in this pathway by BLASTX analyses (Table S1). Overexpression of PI(3)K proteins in the wing or eye imaginal discs in *Drosophila* results in enlarged wings or eyes, respectively, while mutation in this gene results in flies with smaller wings and eyes, which are caused by changes in both cell size and cell number [38]. Additionally, PI(3)K regulates cell division and cell survival in the imaginal discs by controlling the cell number [41]. The gene *DPTEN*, which is human tumor suppressor gene *PTEN* homolog, encodes a putative cytoskeleton-associated molecule with both protein phosphatase and phosphatidylinositol 3,4,5-trisphosphate (PIP3) 3-phosphatase activities. It is known to control cell number and growth by antagonizing PI(3)K, through PI3K-dependent and -independent pathways [38], [42], [43]. The phosphoinositide-3-OH-kinase-dependent serine/threonine protein kinase *Akt* (*protein kinase B*) has been reported to affect cell and imaginal disc size during *Drosophila* development in an autonomous manner [40]. In the past decade, lipid rafts have been considered critical for the proper compartmentalization of insulin signaling in adipocytes. Flotillin-1, originally identified as an integral membrane protein [44], was reported to recruit a complex of tyrosine-phosphorylated Cbl and Cbl-associated protein (CAP) to the lipid rafts, and this recruitment is required for GLUT-4 translocation in response to insulin [45].

### Gene expression profile among the different developmental stages

**Figure 6:**
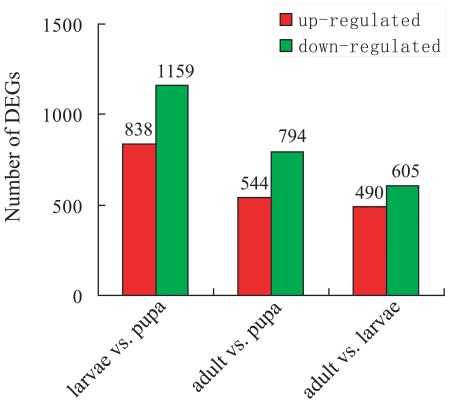
Changes in gene expression profile during development. The number of up-regulated and down-regulated genes between larvae and pupae, between adults and pupae, and between adults and larvae are summarized here.

To identify genes showing differential expression during development, the differentially expressed sequences between two samples were identified (Table S2). There were 2,977 significantly differentially expressed genes detected between the larval and pupal samples, including 838 up-regulated genes (FDR<0.001) and 1,159 down-regulated genes (FDR<0.001, Figure 6 & Figure S2A). The large number of differentially expressed genes between these two samples may be attributed to the important molting and metamorphosis processes during the transition from larva to pupa. A cascade of physiological processes occurs during molting and more complicated physiological processes take place during metamorphosis including histolysis of larval tissues, remodeling and formation of adult tissues, and a molting cascade similar to the larval molt [46], [47]. Additionally, a total of 1,621 differentially expressed genes were detected between adult and larval libraries, with 490 up-regulated genes and 605 down-regulated genes (Figure S2B). Between adult and pupal libraries, 544 genes were up-regulated, while 794 genes were down-regulated, with a total of 2,002 differentially expressed genes (Figure S2C). The number of differentially expressed genes between samples in our transcriptome is different from that of the previously sequenced *B. dorsalis* transcriptome by Illumina [11]. This may be caused by the different samples in these two transcriptomes. The larval stage is limited to the third instar larvae of *B. dorsalis* in the previous transcriptome, while the larval library consists of all larval stages in our new one by 454 pyrosequencing, including the first instar, the second instar and the third instar larvae. Therefore, our new transcriptome makes more detailed and general genetic data available that will facilitate further study of larval development in *B. dorsalis*, especially molting and metamorphosis.

The top 10 most differentially expressed genes between each two samples (larvae vs. pupae, adults vs. larvae, and adults vs. pupae) were analyzed (Table S3). The results showed that most of the upregulated genes in larvae and pupae had orthologs with *Drosophila* and other insects, including those encoding the cuticle protein, the fat body protein, trypsin, and the Ras-related protein. However, nearly 50% of the genes upregulated in adults had no known orthologs in the NCBI database. These findings might be attributed to the fact that developmentally related genes have been more widely identified in *Drosophila* and other Dipteran species than adult genes.

### Functional annotation of differentially expressed genes

To understand the functions of DEGs and identify DEGs involved in signal transduction and important physiological processes, such as development and reproduction, all of the DEGs were mapped to terms in the KEGG database and compared with the whole transcriptome background (Table S4). Among all of the genes with KEGG pathway annotation, a total of 547 differentially expressed genes were found between the larval and pupal stages. Most of them were identified to be involved in metabolic pathways, including oxidative phosphorylation, the citric acid cycle (TCA cycle), and glycolysis/gluconeogenesis. This observation is consistent with that of the previously sequenced *B. dorsalis* transcriptome and suggests that the metabolic rate of the *B. dorsalis* larvae is different from that of the pupa. Between the adult and pupal stages, 416 differentially expressed genes were annotated with KEGG pathway annotation. In addition to oxidative phosphorylation and the TCA cycle, starch and sucrose metabolism-associated genes were also enriched. Adult *B. dorsalis* primarily feeds on sucrose; thus, genes involved in sucrose metabolism are upregulated to adapt to food sources. As for the pathways enriched between the adult and larval stages, 337 differentially expressed genes were identified. Among these, the insulin signaling pathway was notably enriched, which might be attributed to the vigorous growth in the larval stage.

### Verification of differentially expressed genes

**Figure 7:**
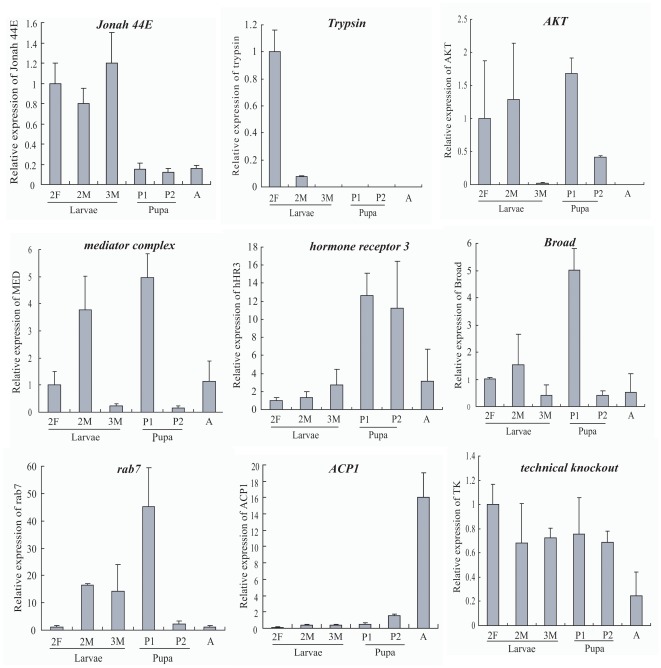
qRT-PCR confirmation of the differentially expressed genes between each two stages (larvae vs. pupae, adults vs. larvae, and adults vs. pupae). The transcript abundance from DEG data is shown above each gene. Relative transcript levels are calculated by real-time PCR using 16 s rRNA as the standard. 2F, feeding 2th instar larvae; 2M, molting 2th instar larvae; 3M, metamorphic molting larvae; P1, prepupa; P2, pupa. Three biological replicates were performed, and the data shown are typical results.

To further evaluate our DEG library, the expression level of 9 genes mostly involved in development and reproduction were analyzed by qRT-PCR. Results showed that real time PCR revealed the same expression tendency as the DEG data, despite some quantitative differences in expression level (Table 4, Figure 7). The effector genes *trypsin* and *Jonah 44E* were highly expressed at the larval stage. Other two genes belonging to the nuclear receptor superfamily, *Broad* and *hormone receptor 3*(*HR3*), were expressed at a high level at the pupal stage. *Broad* is a well-known molecular marker of pupal commitment and has been reported to be involved in mediating the ‘status quo’ action of juvenile hormone on the pupal-adult transformation in *Drosophila* and *Manduca* [48]. *Broad* is abundantly expressed during the formation of the pupa but not in adult differentiation [34], [48]. This is consistent with our result. A member of the small GTPase subfamily, *rab7*, was also expressed abundantly at the pupal stage. Notably, both the *serine/threonine-protein kinase AKT* and *mediator complex* (*MED*) were highly expressed in both larvae and pupae. This finding is consistent with their roles in controlling cell and imaginal disc size during development in *Drosophila* [40]. An effector gene *adult cuticle protein 1* (*ACP1*) was expressed significantly at the adult stage, but its specialized cuticle function needs further study. As a control, *technical knockout* from the non-DEGs library was demonstrated to be equally expressed during different developmental stages. This will provide us with more molecular targets for further study of development and reproduction in *B. dorsalis* and other Tephritid species.

**Table 4:** Comparisons of the DEGs data and qRT-PCR results.

Gene	Gene ID	DEGs library	fold by DEG	P value	Q value	fold by qPCR
*Jonah 44E*	Contig10080	larvae vs. pupa	6.47*	3.47E-11	8.43E-10	6.72
		adult vs. larvae	−5.39**	1.75E-08	3.15E-07	−6.12
*Trypsin*	Contig4994	larvae vs. pupa	7.26	1.31E-17	5.50E-16	230.01
		adult vs. larvae	−6.18	1.12E-13	3.39E-12	−223.53
*AKT*	Contig8031	larvae vs. pupa	−2.41	5.09E-06	5.82E-05	−1.52
*MED*	Contig10114	adult vs. larvae	−5.35	2.73E-08	4.82E-07	−3.90
*HR3*	Contig25289	adult vs. pupa	−4.32	8.18E-05	0.000995	−4.17
		larvae vs. pupa	−5.18	4.82E-06	5.57E-05	−4.20
*Broad*	Contig9781	larvae vs. pupa	−1.66	1.79E-06	2.26E-05	−3.32
		adult vs. larvae	−4.31	0.000184	0.001534	−3.10
		adult vs. pupa	−5.97	4.08E-13	1.72E-11	−8.03
*Rab7*	Contig21195	adult vs. pupa	−5.13	6.75E-08	1.53E-06	−13.23
		larvae vs. pupa	−2.67	1.34E-06	1.73E-05	−2.60
*ACP1*	Contig234	adult vs. larvae	11.96	0.00	0.00	40.32
		adult vs. pupa	9.37	0.00	0.00	15.06
*TK*(control)***	Contig13751	larvae vs. pupa	0.80	0.54	0.68	/
		adult vs. larvae	−0.72	0.63	0.81	/
		adult vs. pupa	0.08	0.96	0.99	/

6.47*: up-regulated at larval stage (P value<0.001);

−5.39**: down-regulated at adult stage (P value<0.001);

*TK* (control) ***: non-DEG.

### Conclusion

We have generated a comprehensive transcriptome of the *B. dorsalis* during development using a 454 FLX platform. The single run produced 28,782 contigs with a mean size of 750 bp. A great number of genes involved in reproduction, development and most major signaling pathways were found in this transcriptome. Additionally, genes differentially expressed at different developmental stages were largely identified and functionally annotated with KEGG database. To our knowledge, this is the first report using 454 sequencing technology for a Tephritid fruit fly species lacking a reference genome. These data make a substantial contribution to existing sequence resources for the oriental fruit fly, provide many more potential molecular targets for *B. dorsalis* control, and may aid in studies of the mechanisms of development and reproduction in fruit flies.

## Methods

### Insect culture and sample collection

*B. dorsalis* were cultured in our laboratory at 28°C under a 12 h light∶ 12 h dark photoperiod. Adult flies were reared on artificial diets (25% yeast extract and 75% sugar) and oviposited into bananas. The hatched larvae fed inside according to the methods described by Li et al. [49]. Larvae of three instars were collected as one sample. The pupae from different stages (prepupa, new pupa, mid pupa, and old pupa) were collected as another sample. Newly emerged adults and sex matured adults before and after copulation were collected as the third sample (the sex ratio was 1∶1, Table 1). At least ten insects were collected for each stage.

### RNA extraction, mRNA purification, and cDNA synthesis

Each frozen sample was ground in mortars with liquid nitrogen, and then total RNA was isolated using TRIzol reagent (Invitrogen) following the manufacturer's instructions. The concentration of total RNA was determined using the NanoDrop (Thermo Scientific, USA), and the RNA integrity value (RIN) was checked using the RNA 6000 Pico LabChip on an Agilent 2100 Bioanalyzer (Agilent, USA). For mRNA purification, total RNA was incubated with 10 U DNase I (Ambion) at 37°C for 1 h, followed by a purification step using the MicroPoly (A) Purist Kit (Ambion) according to the manufacturer's instructions. Then, the purified mRNA was dissolved in the RNA storage solution, and the final concentration was determined using the NanoDrop.

Double-stranded cDNA was synthesized from mRNA according to Ng's full-length cDNA synthesis protocol with some modifications [50]. A GsuI-oligo dT primer was used for the first-strand cDNA synthesis with 10 µg of mRNA and Superscript II reverse transcriptase (Invitrogen). After incubation at 42°C for 1 h, the 5′-CAP structure of mRNA was oxidized by NaIO_4_ (Sigma) and ligated to biotin hydrazide, which was used to select complete mRNA/cDNA heterodimers by binding Dynal M280 beads (Invitrogen). After the second strand cDNA synthesis, the polyA and 5′ adaptor was removed by GsuI digestion.

### cDNA sequencing

The cDNA was fractioned ultrasonically using cDNA size fractionation columns (Agencourt, USA). Each cDNA fraction found to be larger than 800 bp was sonicated to the range of 300–800 bp, and then pooled together with the other cDNA samples ranging from 300 bp to 800 bp.

The prepared cDNAs were transformed into single-stranded template DNA (sstDNA) libraries with the GS DNA Library Preparation kit (Roche Applied Science). The sstDNA libraries were clonally amplified in a bead-immobilized form with the GS emPCR kit (Roche Applied Science) and sequenced on a 454 Genome Sequencer FLX instrument.

### Sequence assembly

Raw reads were firstly cleaned by removing adaptor sequences and low quality sequences (reads with unknown sequences ‘N’), and then assembled into EST clusters (contigs) using CAP3 with the default assembly parameters. The unassembled reads were considered as singlets. The raw data from 454 reads have been deposited into the NCBI Short Read Archive under the accession no. SRA047953.

### Sequence annotation

All contigs and singlets were annotated with GetORF from the EMBOSS package [51]. The ORF of each predicted protein was used for BLASTp searches against the Swiss-Prot and the NCBI nr databases setting the e-value threshold to 10^−4^. GO annotations were also derived based on sequence similarity with GoPipe [52]. Predicted proteins were first used for BLASTp against the Swiss-Prot and TrEMBL databases using an E-value cut-off of 10^−4^. GO annotations were analyzed by GoPipe according to the gene2go software. The COG and KEGG pathways annotations were performed using Blastall software against the Cluster of Orthologous Groups database and the Kyoto Encyclopedia of Genes and Genomes database [53]. In this study, we used the default parameters in each approach and no other custom approach was used.

### Analysis of differentially expressed genes (DEGs)

To analyze genes differentially expressed at the different developmental stages, the number of reads for each of the contigs from the three samples was converted to Reads per Kilobase per Million (RPKM) [54]. Then, the MARS model (MA-plot-based method with Random Sampling model) in the DEGseq package was used to calculate the expression abundance of each contig between the three samples. We used an FDR (false discovery rate) to determine the threshold of p value for this analysis. An FDR<0.001 was considered to have significant expression abundance. For the identification of the pathways that the DEGs were predicted to participate in, we mapped all DEGs to terms in the KEGG database and looked for significantly enriched KEGG terms compared to the genomic background.

### Quantitative real-time PCR (qRT-PCR) verification

9 genes were chosen for the confirmation of DEG data by qRT-PCR using the SYBR Premix Ex Taq kit (Takara, Japan) according to the manufacturer's instructions with a real-time thermal cycler (Bio-Rad, Hercules, CA). TRIzol reagent (Invitrogen, USA) was used to extract total RNA from the *B. dorsalis* at 6 different typical developmental stages (Table 1), including the second instar larvae (feeding and molting larvae), third instar larvae (metamorphosing larvae), prepupae (48 h before pupation), pupae, and adults. At least 10 insects were collected for each sample. The first strand cDNA was obtained from 2 µg of total RNA using M-MLV Reverse Transcriptase (Takara, Japan) with the primer oligo-anchor R (5′-GACCACGCGTATCGATGTCGACT_16_ (A/C/G)-3′). The primers used for qRT-PCR detection of selected DEGs are listed in Table S5. The relative gene expression data were analyzed using the 2^−ΔΔCt^ method as described by Ren et al. [55]. The results were analyzed using a one-way analysis of variance (ANOVA) statistical test. All quantitative PCR were repeated in three biological and three technical replications.

## Supporting Information

Figure S1 Read length distribution from each samples: larva (A), pupa (B) and adult (C). The x-axis shows read size and the y-axis shows the number of reads for each given size.
(TIF)

Figure S2 Comparison of sequence expression between the larvae and the pupae (A), adults and larvae (B), as well as adults and pupae (C). The abundance of each gene was normalized as Reads Per Million (RPM) and Reads Per Kilobase per Million (RPKM). The differentially expressed genes are shown in red and green, while the other genes that are not differentially expressed (not DEGs) are shown in blue.
(TIF)

Table S1 Selected signaling pathway genes identified in the *B. dorsalis* transcriptome with best-hit matches to other insects.
(DOC)

Table S2 Abundance of each contig in the three samples (larva, pupa and adult). RPM, RPKM, and the fold number from each comparison group (larvae vs. pupae, adults vs. larvae, and adults vs. pupae) are shown.
(XLS)

Table S3 The top 10 most up-regulated and down-regulated genes between samples (larvae vs. pupae, adults vs. larvae, and adults vs. pupae).
(XLS)

Table S4 The top 10 most abundantly differentially expressed signaling pathways from each comparison group (larvae vs. pupae, adults vs. larvae, and adults vs. pupae).
(XLS)

Table S5 Primers used for qRT-PCR verification of DEG data.
(DOC)
