# Supplementary figures and images for: High-Throughput Sequencing to Reveal Genes Involved in Reproduction and Development in Bactrocera dorsalis (Diptera: Tephritidae)
Source: PLoS One. 2012 May 3;7(5):e36463. doi: 10.1371/journal.pone.0036463 (PMC3343016; doi:10.1371/journal.pone.0036463)

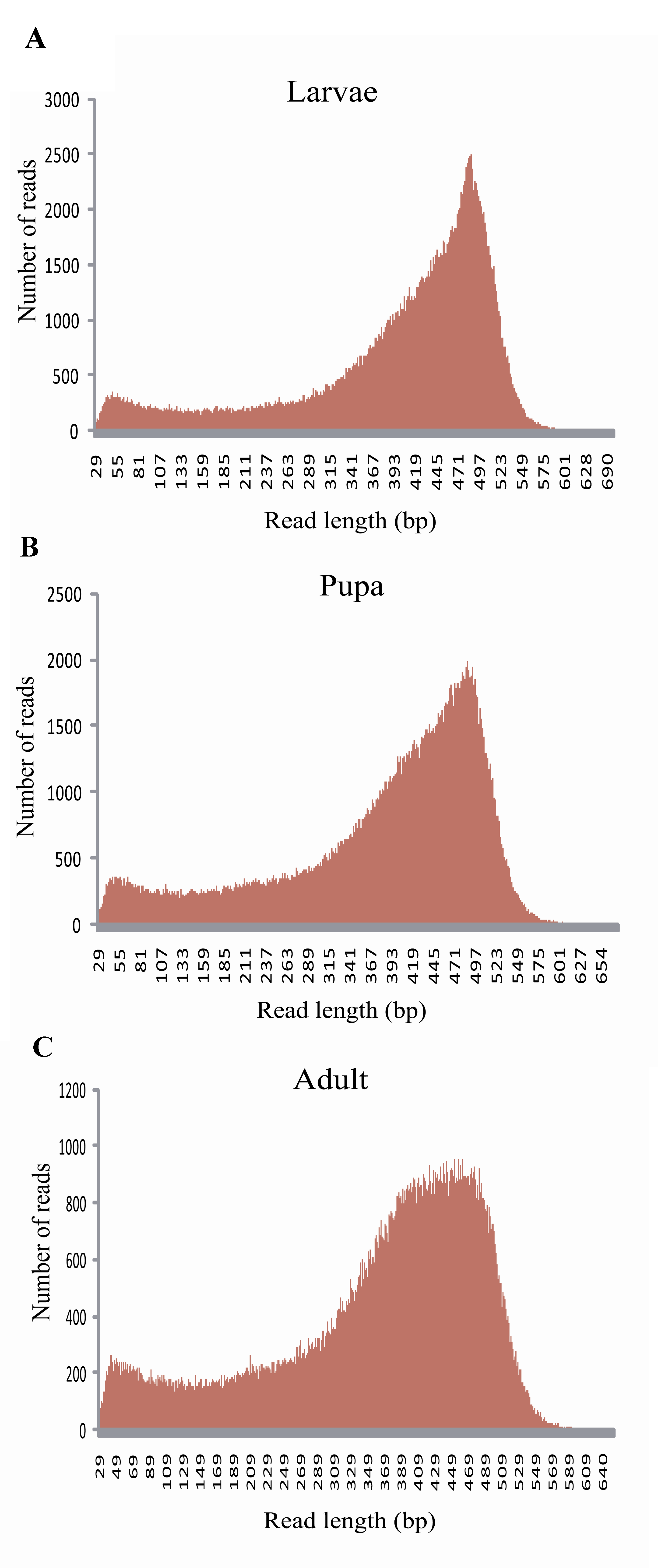

Supplement: Figure S1 — Read length distribution from each samples: larva (A), pupa (B) and adult (C). The x-axis shows read size and the y-axis shows the number of reads for each given size. (TIF) [file pone.0036463.s001.tif]

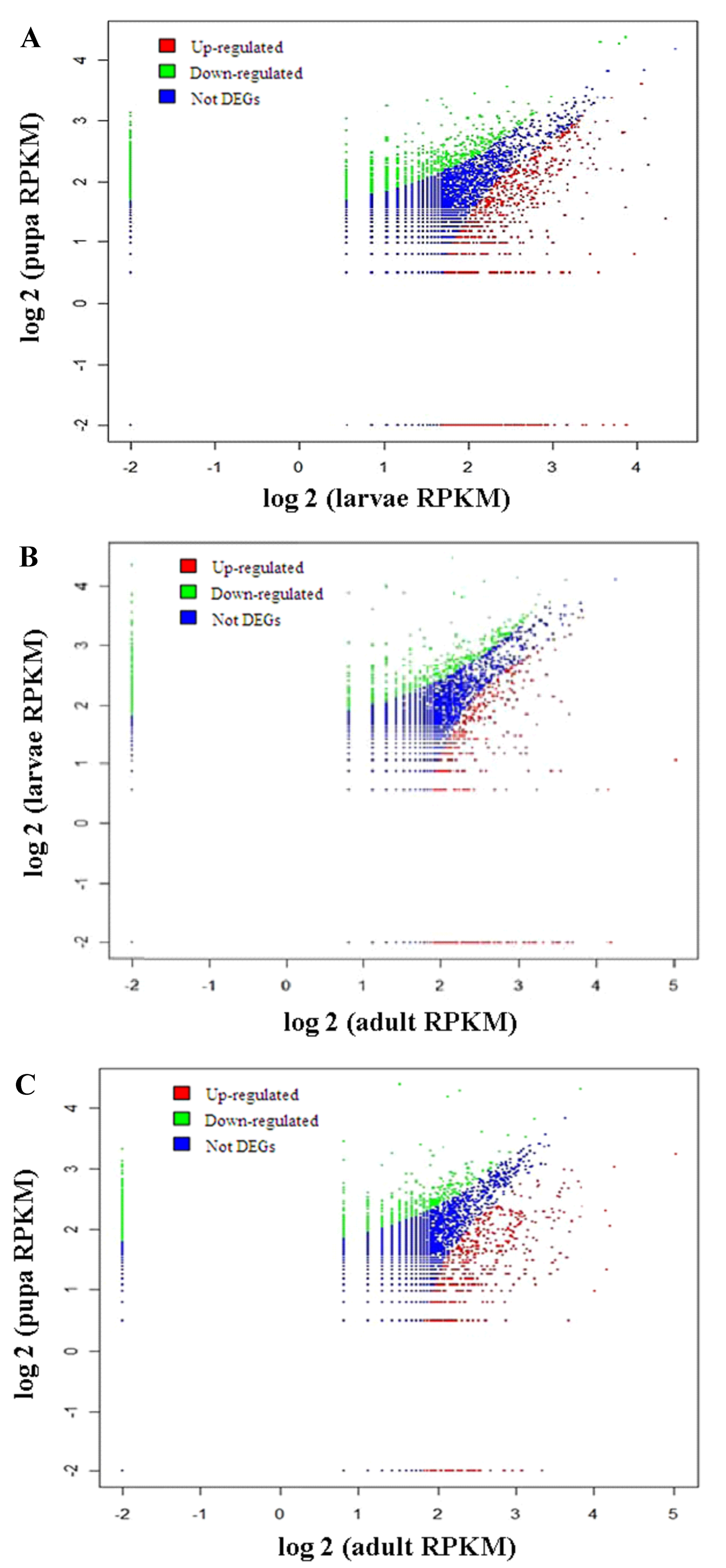

Supplement: Figure S2 — Comparison of sequence expression between the larvae and the pupae (A), adults and larvae (B), as well as adults and pupae (C). The abundance of each gene was normalized as Reads Per Million (RPM) and Reads Per Kilobase per Million (RPKM). The differentially expressed genes are shown in red and green, while the other genes that are not differentially expressed (not DEGs) are shown in blue. (TIF) [file pone.0036463.s002.tif]
