# Supplementary material for: High-Throughput Sequencing to Reveal Genes Involved in Reproduction and Development in Bactrocera dorsalis (Diptera: Tephritidae)
Source: PLoS One. 2012 May 3;7(5):e36463. doi: 10.1371/journal.pone.0036463 (PMC3343016; doi:10.1371/journal.pone.0036463)
Supplement: Table S1 — Selected signaling pathway genes identified in the B. dorsalis transcriptome with best-hit matches to other insects. (DOC) [file pone.0036463.s003.doc]

Table S1 Selected signaling pathway genes identified in the *B. dorsalis* transcriptome with best-hit matches to other insects

| **Pathway** | **Gene ID** | **Length**  **(bp)** | **Subject ID** | **Species** | **E-value** | **Protein**  **Identity (%)** |
| --- | --- | --- | --- | --- | --- | --- |
| **Hormone signaling** |  |  |  |  |  |  |
| *ecdysone receptor* | Contig5526 | 1647 | CAA11907 | *C. capitata* | 0 | 96 |
| *E74* | Contig26070 | 2015 | AAA28493 | *D.melanogaster* | 9e-78 | 89 |
| *hormone receptor 3* | Contig25289 | 1513 | P31396 | *D.melanogaster* | *0* | *91* |
| *ecdysoneless* | Contig24692 | 645 | NP_647707 | *D.melanogaster* | 3e-65 | 56 |
| *ecdysone-induced gene 71E* | Contig25944 | 420 | NP_524089 | *D.melanogaster* | 5e-07 | 35 |
| *broad* | Contig9781 | 2436 | NP_726749 | *D.melanogaster* | 1e-116 | 63 |
| *without children* | Contig27929 | 645 | NP_001097946 | *D.melanogaster* |  |  |
| **Insulin signaling** |  |  |  |  |  |  |
| *PIK3R* | Contig9440 | 638 | NP_477270 | *D.melanogaster* | 4e-26 | 71 |
| *AKT* | Contig8031 | 3193 | NP_732113 | *D.melanogaster* | *0* | *90* |
| *GRB2* | Contig13318 | 1513 | XP_001842429 | *C.quinquefasciatus* | 2e-121 | 92 |
| *IGF2R* | Contig22821 | 737 | XP_967483 | *T.castaneum* | 2e-11 | 31 |
| *insulin-like peptide 1* | 2-G0EDD0L01C3LX9 | 532 | CBY85558 | *D.melanogaster* | 7e-10 | 33 |
| *RAPTOR* | 1-G0EDD0L01AVLRX | 445 | NP_572294 | *D.melanogaster* | 1e-43 | 53 |
| *PTEN* | 2-G0EDD0L01D3763 | 443 | NP_599147 | *D.melanogaster* | 2e-73 | 78 |
| *son of sevenless* | Contig14739 | 702 | AAN61386 | *D.melanogaster* | 2e-48 | 52 |
| *JNK* | Contig28672 | 2580 | AAB51187 | *D.melanogaster* | 0 | 84 |
| *calmodulin* [*.*](http://www.ncbi.nlm.nih.gov/nuccore/24652936) | 1-G0EDD0L01DWQ2X | 551 | NM_078986 | *D.melanogaster* | 8e-98 | 100 |
| *hormone-sensitive lipase* | 1-G0CLBUI04IW844 | 309 | EHJ73918 | D.plexippus | 7e-13 | 57 |
| *flotillin* | Contig9658 | 1697 | NP_477358 | *D.melanogaster* | 0 | 94 |
| *hexokinase* | Contig6583 | 3749 | ADD20426 | G.morsitans | 0 | 68 |
| *eIF-4E* | 3-G0EDD0L02H2DU2 | 406 | NP_729480 | *D.melanogaster* | 8e-104 | 71 |
| *TSC1* | Contig16721 | 632 | NP_477415 | *D.melanogaster* | 4e-40 | 59 |
| *TSC2* | 1-G0EDD0L01DRQN1 | 292 | AAR97337 | *D.melanogaster* | 2e-36 | 66 |
| **WNT** |  |  |  |  |  |  |
| *WNT inhibitory factor 1* | Contig10163 | 724 | XP_001845334 | *C.quinquefasciatus* | 2e-116 | 62 |
| *frizzled 4* | Contig23089 | 1584 | NP_511068 | *D.melanogaster* | 6e-75 | 42 |
| *frizzled 2* | Contig770 | 957 | NP_524155 | *D.melanogaster* | 2e-165 | 78 |
| *disheveled* | Contig1489 | 2443 | NP_511118 | *D.melanogaster* | 0 | 79 |
| *dishevelled associated activator of morphogenesis* | 2-G0EDD0L01BBFMV | 478 | NP_726724 | *D.melanogaster* | 2e-51 | 76 |
| *dally* | Contig16947 | 1205 | AAA97401 | *D.melanogaster* | 2e-84 | 57 |
| **MAPK** |  |  |  |  |  |  |
| *torso-like protein* | 2-G0EDD0L01DBO8R | 492 | NP_524440 | *D.melanogaster* | 1e-82 | 70 |
| *pole hole* | Contig22506 | 813 | NP_001036258 | *D.melanogaster* | 3e-93 | 61 |
| *epidermal growth factor receptor* | Contig2421 | 1174 | XP_001648893 | *A. aegypti* | 0 | 69 |
| *bride of sevenless* | 1-G0EDD0L01BEH53 | 489 | ABB29500 | *D. orena* | 3e-70 | 77 |
| **Notch** |  |  |  |  |  |  |
| *notch* | 1-G0EDD0L01BNOKB | 449 | AAB59220 | *D.melanogaster* | 4e-76 | 82 |
| *strawberry notch* | 1-G0EDD0L01ARPIG | 354 | NP_001096968 | *D.melanogaster* | 2e-67 | 92 |
| *fringe* | Contig24362 | 1091 | NP_524191 | *D.melanogaster* | 0 | 84 |
| *hairless* | Contig15254 | 729 | CAB38221 | *D. hydei* | 2e-19 | 58 |
| *groucho* | Contig11082 | 1402 | NP_733133 | *D.melanogaster* | 2e-124 | 86 |
| *mastermind* | Contig19640 | 840 | AAC37201 | *D. virilis* | 2e-62 | 71 |
| *nicastrin* | Contig16732 | 1655 | NP_99629 | *D.melanogaster* | 8e-170 | 56 |
| *presenilin 1* | Contig3496 | 2039 | AAB53369 | *D.melanogaster* | 0 | 69 |
| **Hedgehog** |  |  |  |  |  |  |
| *smoothened* | 2-G0EDD0L01C5KYM | 499 | NP_523443 | *D.melanogaster* | 6e-69 | 71 |
| *fused* | 3-G0EDD0L02IM8QR | 463 | AAW47574 | *D. ezoana* | 6e-93 | 84 |
| *casein kinase 1* | Contig849 | 882 | NP_511140 | *D.melanogaster* | 6e-145 | 89 |

*C. capitata: Ceratitis capitata*; *T. castaneum: Tribolium castaneum*; *C. quinquefasciatus Culex quinquefasciatus; D. plexippus: Danaus plexippus*; *D. orena: Drosophila orena*; *D. hydei: Drosophila hydei*;

*D. virilis: Drosophila virilis*; *D. ezoana: Drosophila ezoana*
