# Supplementary material for: High-Throughput Sequencing to Reveal Genes Involved in Reproduction and Development in Bactrocera dorsalis (Diptera: Tephritidae)
Source: PLoS One. 2012 May 3;7(5):e36463. doi: 10.1371/journal.pone.0036463 (PMC3343016; doi:10.1371/journal.pone.0036463)
Supplement: Table S5 — Primers used for qRT-PCR verification of DEG data. (DOC) [file pone.0036463.s007.doc]

Table S5 Primers used for qRT-PCR verification of DEG data.

| Primer name | (5′→3′) nucleotide sequence |
| --- | --- |
| Jonah44ERTF | TTGGTTGGGTGGCATACTTC |
| Jonah44ERTR | CCATTCCGTCTCCTACTCG |
| TrypsinRTF | ACACCAACCACCATCAGTTC |
| TrypsinRTR | ACACCACCAGAGTTCCAGTAG |
| AKTRTF | TGGGCAAGATGATGTCAAAG |
| AKTRTR | ATCGGACACTACTTGTGGTTTA |
| MEDRTF | TAATGGAAATCTTATGGACGAG |
| MEDRTR | GCCGTGCAACATCAATAAACC |
| HR3RTF | GCGTGAGAAAGTGGAGGAA |
| HR3RTR | CCCATAACCGTAGGGACTG |
| BroadRTF | AATGGCAACTCGGACACTG |
| BroadRTR | GCTAGATCGGTTCGCTTCA |
| Rab7RTF | AGCGTTGGAAAGACCTCA |
| Rab7RTR | ATCAGCGCCACGATAAAA |
| ACP1RTF | GATTGAGGATTGAACACCCAT |
| ACP1RTR | CGGTCAAGTACAATTCGCTTA |
| TKRTF | CCGCACTGGTCCACATTA |
| TKRTR | GCGAGCATTTCTTTACCG |
| 16srRNARTF | CTCGTCCAACCGTTCATACC |
| 16srRNARTR | CTGACCTGCCCACTGAAGTT |
